# A Case of Stricture and Perforation of the Colon

**Published:** 1826-07

**Authors:** 


### STRICTURE AND PERFORATION OF THE COLON.

A woman, 71 years of age, was received into the Infirmary of the Salpetriere on the 11th October, 1825, who could give no satisfactory history of her complaint. She was pale, emaciated, belly distended and painful on pressure; it was also sonorous on percussion—vomiting from time to time—pulse full and quick—skin hot and dry. Lavements, fomentations, and acidulated drink. This state continued during eight days, without any material change. The patient passed no stools during that period. On the 19th they began to think there were some fæcal matters vomited up; and on the 20th there was no doubt of the fact. No passage through the bowels. From the 20th till the 24th, the patient continued to vomit dark-coloured matters, and death put a period to her sufferings on the morning of the 25th October.

*Dissection.* The abdomen was enormously distended—as were the convolutions of the intestines themselves, when the peritoneum was laid open. This distention disappeared at the commencement of the rectum, which was found contracted and wasted, so as to be scarcely traceable in the cavity of the pelvis. The colon was greatly distended, and the vessels of the intestines and mesentery immensely gorged. The peritoneum was healthy. In the left iliac fossa and in the pelvis was found a quantity of matters similar to those which had been vomited up. In the sigmoid flexure of the colon was found an ulcer, about nine lines in breadth, the bottom of which had penetrated the peritoneal coat, and permitted the exit of the contents of the gut. At the beginning of the rectum there was a stricture, which would scarcely admit a goose-quill, caused by a scirrhous thickening of the mucous and muscular coats of the intestine. This stricture occupied only a small space of about half an inch, above and below which, the coats were sound. From this stricture up to the stomach itself, the intestinal canal was greatly distended, and filled with fæcal matters and gas.—*M. Billard. Bibliotheque, Janv.* 1826.

